# Molecular dynamics studies reveal structural and functional features of the SARS‐CoV‐2 spike protein

**DOI:** 10.1002/bies.202200060

**Published:** 2022-07-17

**Authors:** Ludovico Pipitò, Roxana‐Maria Rujan, Christopher A. Reynolds, Giuseppe Deganutti

**Affiliations:** ^1^ Centre for Sport, Exercise and Life Sciences (CSELS) Faculty of Health and Life Sciences Coventry University Coventry UK

**Keywords:** drug repurposing, epitope, glycans, molecular dynamics, SARS‐Cov‐2, spike protein, variant

## Abstract

The SARS‐CoV‐2 virus is responsible for the COVID‐19 pandemic the world experience since 2019. The protein responsible for the first steps of cell invasion, the spike protein, has probably received the most attention in light of its central role during infection. Computational approaches are among the tools employed by the scientific community in the enormous effort to study this new affliction. One of these methods, namely molecular dynamics (MD), has been used to characterize the function of the spike protein at the atomic level and unveil its structural features from a dynamic perspective. In this review, we focus on these main findings, including spike protein flexibility, rare S protein conformational changes, cryptic epitopes, the role of glycans, drug repurposing, and the effect of spike protein variants.

## Graphical abstract

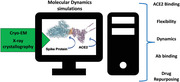

## INTRODUCTION

The year 2019 signaled the start of the worldwide outbreak of Coronavirus disease (Covid‐19)^[^ ^1^, ^2^, ^3^ ^]^ from the *Coronaviridae* SARS‐CoV‐2 virus, which counts around 360 million cases around the world with more than 5.6 million certified deaths (WHO dashboard, January 27, 2022). *Coronaviridae* is an enveloped positive‐stranded, non‐segmented RNA virus with a genome of about 30 kb.^[^ ^4^ ^]^ *Coronaviridae* viruses are responsible for cardiovascular, hepatic, respiratory, gastrointestinal, and neurological diseases, with major symptoms associated with a hyperbolic expression of proinflammatory signals and cytokines such as interleukins, interferon‐gamma (IFN‐γ), IFN‐γ induced protein 10 (IP‐10), macrophage inflammatory protein 1A and 1B (MIP‐1A, MIP1‐B), platelet‐derived growth factor (PDGF), tumor necrosis factor (TNF‐α), and vascular endothelial growth factor (VEGF).^[^ ^5^ ^]^

**FIGURE 1:**
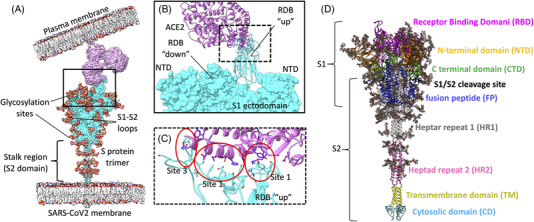
The S protein is the first promoter of SARS‐CoV‐2 internalization. (A) Overall architecture of the complex between S protein (prefusion conformation, cyan) and ACE2 (violet); glycans on S protein are in van der Waals spheres; the relative positions of the plasma and viral membranes are reported. (B) Magnification of the S1 ectodomain (glycans removed for clarity); the RBD in the up conformation is responsible for binding ACE2. (C) Magnification of the interface between the RBD and one of the ACE2 monomers; the interactions can be divided according to the relative position into sites 1 to 3 (red circles). (D) Fully glycosylated S protein (https://charmm‐gui.org/?doc=archive&lib=covid19) with the S1 and S2 units highlighted. The NTD, RBD, CTD, S1/S2 cleavage site, FP, HR1, HR2, TM, and CD are indicated. Glycans are shown in licorice. ACE2, angiotensin‐converting enzyme 2; CD, cytosolic domain; CTD, C‐terminal domain; FP, fusion peptide; HR, heptad repeat; NTD, N‐terminal domain; RBD, receptor‐binding domain; TM, transmembrane domain

The SARS‐CoV‐2 infection mechanism depends on the transmembrane spike protein (S protein, Figure 1A,D),^[^ ^6^, ^7^ ^]^ a highly conserved structure amongst the *coronaviridae* family responsible for extracellular binding and cell membrane fusion.^[^ ^8^ ^]^ It characterizes the shape of this family of viruses, giving it the “solar” crown aspect^[^ ^9^ ^]^ they are named after. The SARS‐CoV‐2 strain shows a selective affinity for the angiotensin‐converting enzyme 2 (ACE2, Figure 1A) receptor, a type 1 transmembrane protein with an external peptidase domain (PD) normally responsible for the conversion of angiotensin hormone into angiotensin II.^[^ ^10^ ^]^

The S protein has aroused the interest of medical and pharmaceutical research to prevent infection and reduce the burden of clinical intervention. It is a homotrimer class I fusion protein, with each protomer composed of domain S1 and S2 (in the prefusion conformation, Figure 1A).^[^ ^11^ ^]^ The S1 structure is responsible for binding ACE2 (Figure 1A–C), before the conformational change in the stalk‐like structure (Figure 1A,D) of the S2 subunit^[^ ^12^ ^]^ and the subsequent membrane fusion after the cleavage of S1 from S2 by the host transmembrane protease serine 2 (TMPRSS2).^[^ ^9^ ^]^ The cleavage of the inter‐region S1/S2 (Figure 1A,D) allows for S2 structural conformation changes necessary for membrane fusion and post‐fusion structure adaptation.^[^ ^8^ ^]^ In the S1 ectodomain (Figure 1D), the apical portion of the S protein, composed of the receptor‐binding domain (RBD), the N‐terminal domains (NTDs), and two C‐terminal domains (CTDs), folds in a hairpin motif that protects the prefusion conformation of S2 from the external environment.^[^ ^13^ ^]^ A distinguishing feature of the SARS‐CoV‐2 strain is an insertion in the protease S1/S2 cleavage site region, rich in arginine, which configures a furin recognition site, commonly found in highly virulent influenza viruses.^[^ ^14^ ^]^

**FIGURE 2:**
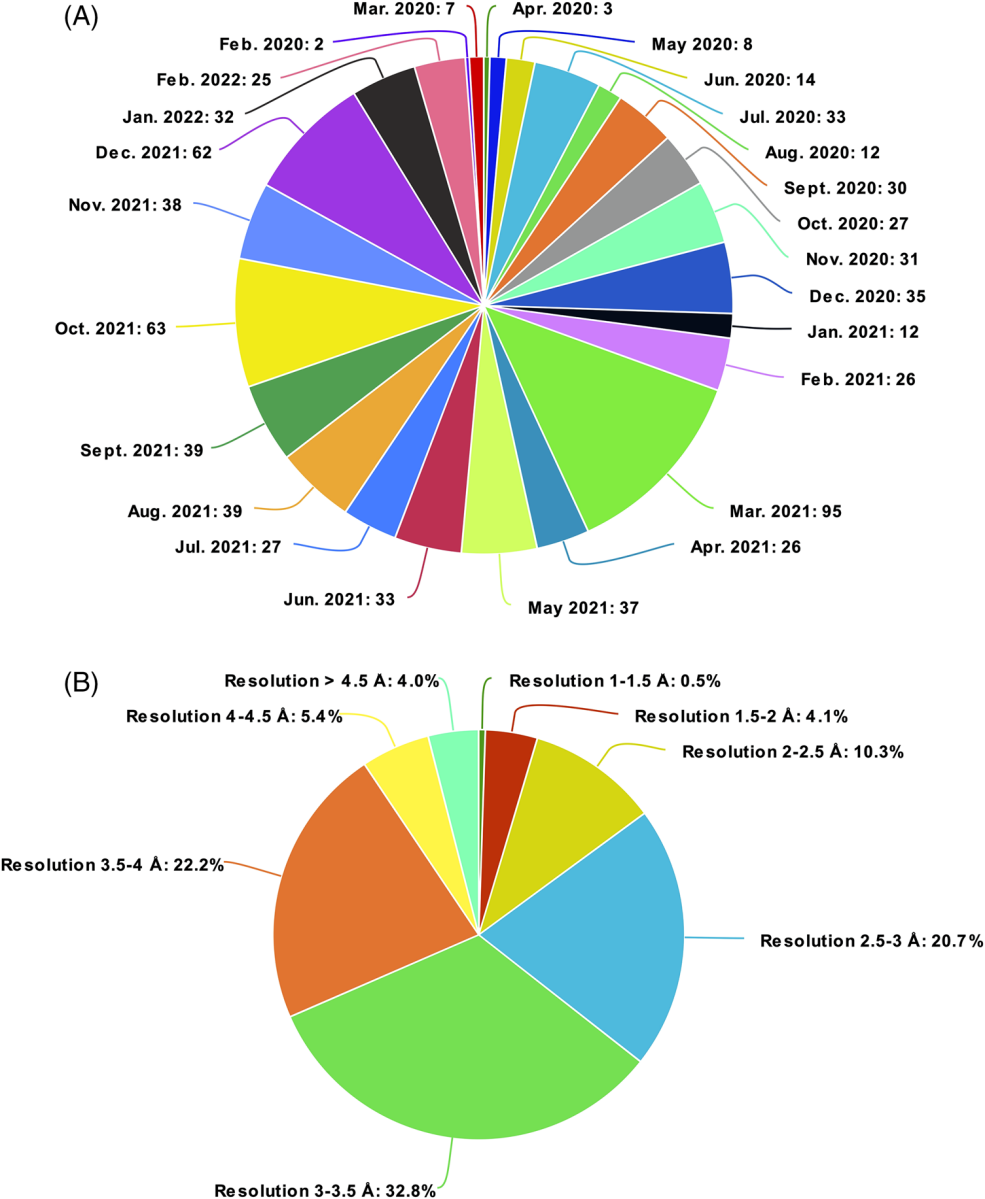
S protein deposited structures in the protein data bank. (A) SARS‐CoV‐2 protein structures released and ordered according to month and year of publication from the Protein Data Bank^[^ ^18^ ^]^ (B) S protein structures only, ordered according to resolution

Several S protein structures have been determined through cryo‐electron microscopy (cryo‐EM) and X‐ray crystallography (Figure 2).^[^ ^15^ ^]^ These include the inner S1 and the external S2 domains and indicate two different states in the RBD domain, named “up” and “down,”^[^ ^11^, ^16^, ^17^, ^18^ ^]^ the former determining an active state^[^ ^19^ ^]^ favorable to ACE2 binding (Figure 1A–C). For S1 to bind ACE2,^[^ ^20^ ^]^ the RBD must undergo a conformational hinge movement, exposing the hydrophobic region between A570‐T572, F855‐N856 at the interface between RBD and S2 in an “up” conformation state.^[^ ^17^, ^21^ ^]^ The *Coronaviridae* family has a distinctive morphology characterized by a spherical virion with a diameter of 91 ± 11 nm measured at the membrane, on whose surface there are 24 ± 9 S trimers unevenly distributed with a prevalence of 97% of trimers in “down” conformation^[^ ^16^ ^]^ at room temperature. Although cryo‐EM studied by Benton et al., showed that only 11% of the total trimeric structures were fully closed, 20% are in the open state either with one RBD (16%) or two RBD up (4%).^[^ ^13^ ^]^ The RBD is responsible for ACE2‐specific binding through an ensemble of 16 well‐conserved residues directly interacting with the receptor^[^ ^22^ ^]^ (Figure 1A–C). Three different sites (Figure 1C), named according to which part of ACE2 they bind, can be distinguished. Site 1 (identified by residue Gln498, Thr500, Asn501, Tyr505) and Site 3 (Asn487 and Phe486) bind to the α1 helix C (Gln24 and Thr27), while Site 2 (Arg403, Tyr453, Leu455, Phe456, and Gln493) binds to the center of the helix (Asp30, Lys31, His34, Asp38) which is slightly bent outwards, exposing polar amino acids for interaction.^[^ ^23^ ^]^ The RBD is an important target for preventing or treating the SARS‐CoV‐2 infection.^[^ ^9^, ^24^ ^]^ A common trait shared among the coronavirus family is the post‐translational N‐ and O‐glycosylation used to mask the S protein epitopes and escape from immune system recognition,^[^ ^25^, ^26^ ^]^ covering approximately 40% of the surface protein, especially N343 which seems to hinder antibody binding. A recent cryo‐EM‐derived S protein model revealed that 44 out of 66 potential sites are heavily *N*‐glycosylated in the ectodomain region (Figure 1A).^[^ ^11^ ^]^

Molecular dynamics (MD) is a computational technique that simulates the evolution over time of (bio)molecular structures. It represents a state‐of‐the‐art tool for biophysical studies and structure‐based drug design^[^ ^23^ ^]^ as it describes the positional changes of the atoms of a chemical system that explicitly includes water, ions, and other biological components such as protein, membrane, and nucleic acids,^[^ ^27^ ^]^ allowing the conformational exploration of biological structures. The evolution over time is simulated by integrating Newton's classical equation of motion for each atom of the system. The result of this many‐particle motion is a trajectory, from which it is possible to extrapolate thermodynamic, kinetics, and physical properties through statistical mechanics models.^[^ ^28^ ^]^ One of the advantages of MD is to overcome the unnatural rigidity that characterizes most X‐ray crystallography and cryo‐EM structures, allowing the investigation of possible cryptic binding pockets, allosteric effects, and structural changes in response to the binding.

In this review, we recapitulate the MD studies that have expanded our knowledge of the SARS‐CoV‐2 S protein flexibility and antibody (Ab) recognition and summarize their contribution to drug repurposing campaigns.

## MOLECULAR DYNAMICS SIMULATIONS UNCOVER THE S PROTEIN FLEXIBILITY

Since the first cryo‐EM structures of the S protein became available to the scientific community, it has been possible to investigate the conformational changes and the dynamic processes involving the S protein through MD simulations. One of the limits of the S protein structure experimentally determined is the scarcity of structural information about the post‐translational glycosylation, due to the high dependency on the organism used for protein expression. To address this aspect, Woo et al. proposed a set of complete and fully glycosylated (Figure 1) S protein models,^[^ ^29^ ^]^ corroborating Wrapp et al.’s structure of the spike protein glycosylated in 44 out of 66 possible sites in the ectodomain region^[^ ^11^ ^]^ including also Watanabe and coworkers glycans’ specifications.^[^ ^30^ ^]^

A study by Turoňová et al.^[^ ^18^ ^]^ as cited by Choi et al.^[^ ^31^ ^]^ showed that the S1 domain displays structural compactness, while the stalk is characterized by two flexible portions, at the heptad‐repeat 2 (HR2) linker and the heptad repeat transmembrane portion (HR2‐TM), respectively. Such findings are in line with the experimental observation that the S protein can tilt up to 90° toward the membrane, with an inclination of 48° (to the membrane normal) being most likely to occur. Such movements might favor ACE2 binding^[^ ^31^ ^]^ by scanning the surrounding space for a possible alignment with the receptor, while indirectly exposing cryptic epitopes.^[^ ^16^ ^]^ These findings, in context with the cryo‐EM results,^[^ ^18^, ^32^ ^]^ highlighted the importance of S2 flexibility, which plays a crucial role in conformational changes,^[^ ^33^ ^]^ alignment, and membrane fusion process.^[^ ^7^, ^16^ ^]^ However, the limitations of the timescale in MD simulations restrict the exploration of long‐lasting contacts between the protein and the receptor, thus limiting our ability to characterize the interaction network that contributes to the binding process.

The sequence of conformational changes on the S2 domain, necessary for membrane fusion, is triggered at the S1/S2 cleavage site on residues Pro681‐Arg684^[^ ^34^ ^]^ and facilitates the exposure of the FP.^[^ ^35^ ^]^ However, the experimental determination of these conformational changes is difficult due to the rapid timescale involved. A computational attempt was made by Remington et al.,^[^ ^36^ ^]^ through the use of nontargeted parallel cascade selection MD (nt‐PaCS‐MD). The variational approach to Markov processes (VAMP) analysis indicated distinct conformational changes in cleaved SARS‐CoV‐2‐spike models at the level of residues Val705–Asp1146 and residues Ser816–Asp1146.^[^ ^36^ ^]^ These changes seemed necessary to expose the fusion peptide (FP) and rearrange the region between residues Ile818–Val826 of the FP into an outward‐facing helical structure that might mechanically initiate membrane fusion, indicating the crucial role of the S1/S2 cleavage site in facilitating the fusion mechanism. These findings suggest that drug discovery could target the S1/S2 cleavage site to hamper an efficient exposition of the FP, therefore interfering with the membrane fusion mechanism.

The differences between SARS‐CoV and SARS‐CoV‐2 were addressed to understand the reasons behind SARS‐CoV‐2's high infectivity and the molecular mechanisms required for effective therapy development. Furthermore, due to the presence of multiple mutations that differentiated SARS‐CoV‐2 and its variants from the original SARS‐CoV, the molecular investigation of residues and conformational differences became necessary for a prompt pharmaceutical response. MD studies of SARS‐CoV‐2 have indicated accentuated flexibility compared to its predecessor SARS‐CoV in segments of the RBD implicated in the molecular recognition of ACE2, more precisely in the region comprising residues Gln474–Gly485, Cys488–Phe490, and Ser494–Tyr505 of the RBD, which also enhances binding to the ACE2 receptor^[^ ^37^ ^]^ in B.1.351 and B.1.1.7 variants. It was possible, through MD simulations in combination with free energy perturbation (FEP), to assess the effect of mutations such as Asn501Tyr and Glu484Lys; the calculations indicated that the binding to ACE2 improved by 4.5 and 1.3 kcal/mol, respectively. The flexibility of RBD in the “up” conformation has been proposed as a determinant for the high propensity of SARS‐CoV‐2 to reach ACE2, giving rise to the high infectivity associated with SARS‐CoV‐2^[^ ^38^ ^]^ compared to SARS‐CoV.^[^ ^37^ ^]^ MD investigations allowed a broader analysis of the interaction network between ACE2 and RBD, which was not observed in the static cryo‐EM or X‐ray crystal structures. MD simulations showed a large interaction network between residues Ile21, Gln24, Thr27, Phe28, Asp30, Glu35, Asp38, Ala80, Met82, and Tyr83 of ACE2 and the RBD.^[^ ^39^ ^]^

A study by Barros et al.^[^ ^40^ ^]^ indicated that ACE2 presents great motility when in contact with the S protein, suggesting that rotation of the catalytic zinc‐binding PD along the transmembrane domain axis could sterically accommodate multiple ACE2 bindings. This large shift appeared to be enhanced by five glycan residues bound to Asn53, Asn90, Asn103, Asn322, and Asn546 of ACE2, with Asn53 involved in both intramolecular homodimer and heterodimer contacts.^[^ ^40^, ^41^ ^]^ The results by Williams et al.,^[^ ^42^ ^]^ in conjunction with those by Barros, indicated that in the RBD‐ACE2 interaction pattern, residues Phe486, Asn487, and Tyr489 are responsible for the adaptive flexibility of the RBD in establishing strong interactions with ACE2. Taken together, these results describe the synergy between a strong ACE2‐binding RBD, which once locked, is carried by the rotation of the ACE2 axis, allowing for multiple receptor engagement and a sequential binding mechanism. At the same time, this study demonstrated how mutations in that RBD sub‐region are crucial in the selective pressure of the virus, altering the flexibility of RBD and interfering in intra‐monomer interactions within the RBD.^[^ ^42^ ^]^ From a geometric perspective, effective interaction between the SARS‐CoV‐2‐spike protein and ACE2 would occur at an angle of inclination between the apical portion of RBD “up” and ACE2 of at least 52°.^[^ ^43^ ^]^ Such MD results indicated that RBD “up” conformations have a large degree of maneuver to achieve sufficient residue exposure for ACE2 binding.

Although MD is able to describe the dynamic events that lead to conformational changes and new interactions, predictions are still limited by the computational cost and represent a simplified scenario, where the complexity of a cellular microenvironment cannot be adequately represented.

## MOLECULAR DYNAMICS SIMULATIONS TO EXPLORE RARE S CONFORMATIONAL CHANGES

The activation of a protein occurs through a series of conformational changes driven by molecular interactions with the intended target. The exploration of metastable states is necessary to understand the intermediate steps occurring during molecular events, and, therefore, identify possible therapeutic targets to interfere with the functional pathway.

However, large protein conformational rearrangements usually take place in the millisecond or second timescale, far beyond the time simulated in MD, which is usually within tens of microseconds. In such a context, the implication of this is that rare conformational changes can be missed. It is, therefore, necessary to apply enhanced or adaptive sampling algorithms to overcome this intrinsic limitation of the sampling to explore drastic structural changes in proteins.

From this perspective, weighted ensemble (WE) MD allows sampling of rare events,^[^ ^44^ ^]^ drastically increasing the computational efficiency. With WE, multiple simulations are run in parallel and the trajectories that explored new values of a metric decided a priori (a distance between atoms in the simplest case) are retained and replicated, thus minimizing the randomness of conformational exploration. By using the WE path‐sampling strategy, Sztain et al.^[^ ^45^ ^]^ were able to simulate the transition state of RBD from “down” to “up,” uncovering the crucial role of several glycan residues in allosterically stabilizing the “up” state. While Asn165 and Asn264 shield the RBD acting as an “up” state stabilizer,^[^ ^46^, ^47^ ^]^ Asn343 pushes the RBD to the final “up” state interacting with residues Phe490, Tyr489, Phe456, and Arg457 on the interaction portion of the ACE2 binding motif.^[^ ^45^ ^]^ More recently, it has been suggested that glycans attached to Asn165 and Asn343 contribute to the overall stability of the RBD open conformation.^[^ ^46^ ^]^

An approach combining WE and artificial intelligence (AI) was adopted by Casalino et al.^[^ ^48^ ^]^ to evaluate transition conformations during the binding between fully glycosylated S protein and ACE2. This confirmed the role that the two *N*‐glycan residues linked to Asn165 and Asn234 have in modulating the dynamics of the S protein's RBD, contributing to the axial mobility of ACE2 while triggering the opening of RBD in a “hand jive” motion. Yao et al.^[^ ^49^ ^]^ analyzed the molecular architecture of SARS‐CoV‐2, from cryo‐electron tomography (cryo‐ET) and subtomogram averaging (STA) highlighting the complex composition of *N*‐glycans, which is the result of unions between branched oligomannose and hybrids units. Such complex glycan ramification also appears to be present on Asn234, whose allosteric role in the conformational change of RBD from “down” to “up” has been demonstrated by Casalino et al.^[^ ^48^ ^]^

**FIGURE 3:**
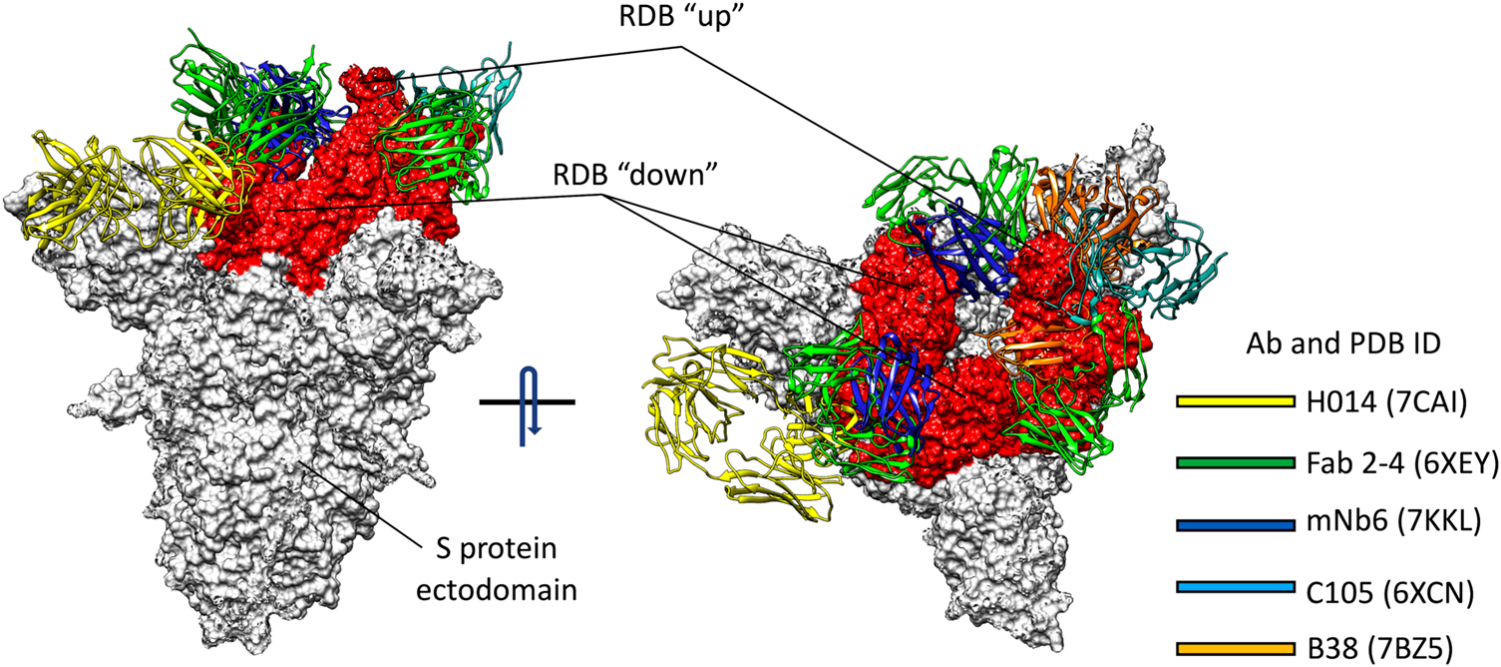
Human antibodies can bind to different S protein epitopes. The binding position of five human Abs on the surface of the S protein, is colored according to the legend. Abs names and protein data bank database IDs are reported in the legend. The S protein is represented as a white surface, with the RBD in red. Abs, antibodies; RBD, receptor‐binding domain

In a separate study, all‐atom steered MD (SMD) forced the RBD from “down” to “up” and highlighted the conformational changes that occur during the breaking of the salt bridges between the RBD and the neighboring protomers, that is, the salt bridges that keep RBD in an inactive “down” state.^[^ ^50^ ^]^ These intramolecular salt bridges, Lys378‐Glu988 and Lys386‐Asp985 within the S2 domain, and Glu516‐Lys202 within the NTD are mainly responsible for the inactive “down” state of the monomers and prevent the interactions with ACE2. Data obtained through targeted molecular dynamics (TMD) have shown how glycans on RBD residues Asn165, Asn234, and Asn343 can act as position lockers for the active “up” conformation,^[^ ^51^ ^]^ stabilizing a set of interdomain salt bridges involving Lys417, Arg408, and Lys378. Furthermore, glycans on Asn165 and Asn234 were proposed to shield the epitopes (Figure 3), while locking the RBD in the “up” state.^[^ ^47^ ^]^

The description of the RBD transition from the down to the up states is a nontrivial task, essential for understanding the protein activation and providing valuable information about cryptic binding pockets. A recent study by Dokainish et al.^[^ ^52^ ^]^ described the opening of RBD by adopting the new generalized replica exchange method with solute tempering of selected surface charged residues (gREST_SSCR), an enhanced method derived from generalized replica exchange with solute tempering (gREST).^[^ ^53^ ^]^ In this study, a selection of charged residues at the RBD interface was regarded as the solute region for gREST, exploring a range of temperatures while the solvent was kept at a constant temperature. The results highlighted the important intra‐chain interaction between R408 (chain A) and the proximal D406 (chain C) and the stabilizing role of the glycan on N165 for the “down” state, while the glycans on N343 and N234 supported the opening of the chain and the stabilization of the “up” state, respectively. The glycan on N343 interacts with Y489 and Q493 in the “up” state, contributing to structural stabilization, with the interdomain contribution of residues S477‐T385, Q493‐C379, Y489‐T385, and Q493‐K378 pairs.^[^ ^51^ ^]^

A remarkable effort was made by Zimmerman et al.^[^ ^54^ ^]^ to explore large conformational changes through Markov‐state models (MSMs) combined with the computational power provided by “citizen‐scientists” from the “Folding@home” project (http://foldingathome.org), very long time scale simulations, in conjunction with the FAST algorithm, described the large conformational changes on the S protein which opened the RBD from its “down” to the “up” state, while the RBD domain twisted outward, exposing new cryptic epitopes.

## HIDE AND SEEK: THE HUNT FOR EPITOPES THROUGH MD

Access to the S protein epitope(s) is necessary for antibody binding (Figure 3).^[^ ^55^ ^]^ From this standpoint, long MD simulations might unveil cryptic epitopes. Sikora et al.^[^ ^55^ ^]^ performed an extensive simulation of four S proteins embedded in a membrane for a total of 2.5 μs. The resulting trajectories were analyzed through simulated illumination analysis and rigid docking of the antibody CR3022. In the illumination analysis, randomly oriented rays emanated from a half‐sphere at the center of mass of the S protein. Rays are then absorbed by the first heavy atom they meet within 1.5 Å. Single S protein structures are collected every 10 ns. To evaluate the shielding effect of glycans, the analysis was replicated without their presence. The results indicated that glycans reduce the S protein accessibility by up to 80%, with the most marked effect occurring in the stalk region close to the viral membrane. An interesting ab initio epitope mapping method was used by Serapian et al.^[^ ^56^ ^]^ Possible epitopes were classified according to the coupling energy with the rest of the structure, which identifies sites on the S protein surface that are at lower binding energy levels and could possibly be energetically preferred bound states. This method suggested that residues forming an epitope prefer to form molecular interactions with external elements.^[^ ^56^ ^]^ The data agrees with the experimentally detected epitope recognized by antibodies CR3022, 4A8, S309, and EY6A.

The use of small molecules or cosolvents as probes in MD simulations (mixMD) increases the chance of discovering cryptic niches or epitopes on the surface of a protein. Through MixMD is possible to map interesting interaction sites by considering the frequency of contacts between protein and probe, resulting in a volumetric map. Using a concentration around 1%–5% cosolvent usually improves the sampling of hot spots for interactions without denaturing the protein. Pyrimidine (Py), acetonitrile, and isopropanol were used to discover possible docking niches on the interface between RBD and ACE2, and to inspire the drug design of antagonists or antibodies.^[^ ^57^ ^]^ Py showed the most relevant volumetric maps within the RBD that spans from residue Gln498 to residue Tyr505. Knowledge of these types of interactions, in conjunction with the molecular mechanics with generalized Born surface area solvation (MM‐GBSA) analysis, leads drug design processes and virtual screening in concordance with experimental data from antibodies, with the data suggesting a set of new molecules (DB02651, DB03714, DB08248, and DB14826) as possible RBD interaction modulators.

## COMPUTER‐AIDED DRUG REPURPOSING TO TACKLE COVID19: THE ROLE OF MOLECULAR DYNAMICS SIMULATIONS

A huge drug repurposing effort (Box 1) was put in place,^[^ ^59^ ^]^ worldwide, to shorten as much as possible the approval of therapeutics against SARS‐CoV‐2 validated targets. Necessarily, the S protein has been one of the most investigated COVID‐19 therapeutic targets due to its unique function and central role in the early stage of infection.

**Box 1**

A possible strategy to overcome the barriers in the research and development of new active compounds is through drug repurposing of existing formulations for a different therapeutic indication (drug repurposing is usually characterized by a substantial variation from its original use). Since the COVID‐19 outbreak, the alarming spread of the virus and the gravity of the infection led the scientific and medical community to seek rapid responses. The general approach to drug repurposing ideally starts with three steps: identifying the candidate molecule to generate the hypothesis, preclinical studies of the candidate molecule, and evaluation of efficacy in phase II clinical trials.^[^ ^58^ ^]^ A wide set of computational techniques and software, which falls under the category of computer‐aided drug design (CADD), is routinely used during the first step of drug repurposing to pinpoint potential drug candidates. CADD includes a plethora of ligand‐based and structure‐based approaches, involving target structural validation, binding energy evaluation in both static and dynamic models, and pharmacokinetics prediction.

The general idea behind targeting the spike protein is to act as a preventive defense against infection, with the intent of minimizing the risk of triggering a potentially dangerous over‐reaction of the immune system, reducing de facto the burden on the public health sector. In May 2020, news about the efficacy against COVID‐19 of hydroxychloroquine (HCQ) originated in China, and the use of HCQ and azithromycin (ATM) was indicated as a possible front‐line treatment. Simulations indicated that HCQ and ATM would have a synergistic effect in the treatment of the infection, where HCQ acts as a competitive binder against gangliosides, another proposed receptor for S protein, and ATM interacts with the tip of SARS‐CoV‐2‐spike.^[^ ^60^ ^]^ Although these results seemed promising, the outcomes of clinical trials appeared highly controversial and the hypothesis of adopting the combined HCQ and ATM therapy has been abandoned.

To face the threat of SARS‐CoV‐2 and its mutations, including the British (alpha) variant,^[^ ^61^, ^62^ ^]^ large companies such as Pfizer BioNTech and AstraZeneca^[^ ^63^, ^64^ ^]^ have developed vaccines capable of activating an immunogenic response against the S protein. A global vaccination campaign has started, with more vaccines currently under development all around the world.^[^ ^65^ ^]^

However, as low‐income countries struggle to have access to vaccines and immunosuppressed and allergic subjects cannot take advantage of the protection offered, alternative therapeutic approaches are still needed. Also, despite the high efficacy of vaccines, the full compliance of the population of high‐income countries is yet to be reached, due to the limited knowledge of the long‐term effects of new mRNA technologies and their implementation.^[^ ^64^ ^]^ In this scenario, drug repositioning could bring many advantages in terms of risk control and unwanted side effect management – because repurposed drugs have already passed safety assessments. Understandably, antiviral agents were among the first agents to be tested against COVID‐19. This approach led to the approval of Remdesivir as the first treatment for hospitalized patients,^[^ ^66^, ^67^, ^68^ ^]^ but not without controversies, due to uncertain outcomes of many clinical trials.^[^ ^67^, ^69^, ^70^ ^]^

Long MD simulations have become a state‐of‐art computational tool in CADD^[^ ^23^ ^]^ as they represent the best tool to validate in silico results of molecular docking and virtual screening campaigns. Here, we report insights from MD simulations applied to the discovery of potential drugs able to interfere with the binding between RBD and ACE2. Only molecules tested both in vitro and in silico are reported.

One of the first computational works on SARS‐CoV‐2 proposed denopamine (Table 1A), bometolol, and Rotigaptide as possible inhibitors of S protein‐ACE2 binding.^[^ ^71^ ^]^ The authors tested denopamine in vitro, observing a diminishing of RBD binding at denopamine concentrations >100 μM.^[^ ^71^ ^]^ An in silico study highlighted simeprevir and lumacaftor as putative RBD binders.^[^ ^72^ ^]^ Lumacaftor (Table 1B) was subsequently proved to weakly bind to S protein with an IC_50_ of 84  ± 4 μM, although showing a good inhibition profile in Vero‐E6 assays.^[^ ^73^ ^]^ Simeprevir (Table 1C) reduces the cellular viral load, synergizing with Remdesivir, but this effect was attributed to a direct action on the main protease and the RNA‐dependent RNA polymerase (RdRp).^[^ ^74^ ^]^ Post‐docking MD simulations identified GSK1838705A, BMS195614, KT185, RS504393 and KT203 (Table 1D–H), five compounds from the Sigma–Aldrich library of pharmacologically active compounds (LOPAC), as potential binders of the S protein.^[^ ^75^ ^]^ A retrospective MD investigation on arbidol (Table 1I), a therapeutic agent approved in China and Russia for influenza, showed an inhibitor effect on the original SARS spike protein^[^ ^76^ ^]^; they proposed arbidol intercalated between different spike protein subunits, and so affecting the trimerization of the S protein.^[^ ^77^ ^]^ Docking and MD simulations performed by ourselves^[^ ^78^ ^]^ and others^[^ ^79^ ^]^ proposed Nilotinib (Table 1J) as a potential binder of the RBD or disruptor of the RBD–ACE2 complex. The anti‐SARS‐CoV potential of nilotinib was first reported in 2016 in the early stages of infection by inhibiting viral fusion at the endosomal level.^[^ ^80^ ^]^ A couple of years later further results pointed out an action of nilotinib and other Abl kinase inhibitors, on the virus‐cell membrane fusion.^[^ ^81^ ^]^ In a recent study, the EC_50_ of imatinib was quantified as 1.44 and 3.06 μM in Vero‐E6 cells and human respiratory cells, respectively.^[^ ^82^ ^]^ Therefore, no experimental evidence for imatinib binding to RBD has been reported. The same goes for nafamostat, which we suggested as a putative RBD binder,^[^ ^78^ ^]^ but it is proposed to act as a TMPRSS2 inhibitor in the low nanomolar range.^[^ ^83^, ^84^ ^]^

**TABLE 1:** Summary of the drugs, recently identified as protective against SARS‐CoV‐2 in vitro, proposed as RBD binders by MD simulations

A 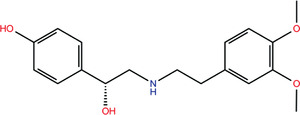	B 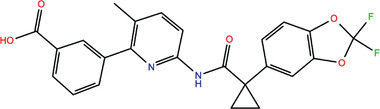
**Denopamine**: cardiotonic drug acting as an agonist at the β1 adrenergic receptor; used in the treatment of angina^[^ ^85^ ^]^	**Lumacaftor**: used for the treatment of cystic fibrosis in patients that present the F508del in the CFTR (cystic fibrosis transmembrane conductance regulator) protein^[^ ^15^ ^]^; IC_50_ of 84 ± 4 μM toward the S protein
C 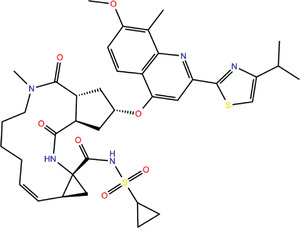	D 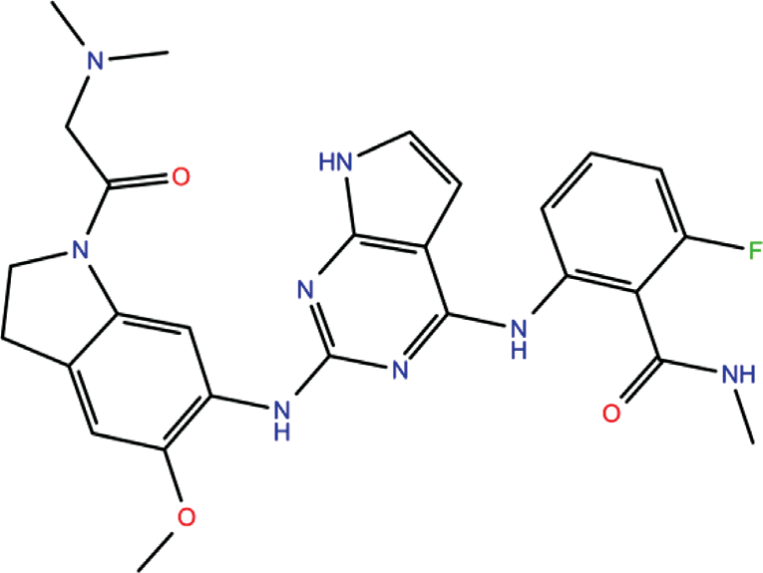
**Simeprevir**: inhibitor of the hepatitis C virus (HCV) NS3/NS4A protease^[^ ^71^, ^87^ ^]^ IC_50_ of 9.6 ± 2.3 μM toward the M^pro^ and an IC_50_ value of 5.5 ± 0.2 μM toward the RdRp (RNA‐dependent RNA polymerase)^[^ ^74^ ^]^	**GSK1838705A**: inhibitor of the insulin‐like growth factor‐1 receptor (IGF‐IR), insulin receptor and anaplastic lymphoma kinase (ALK)^[^ ^88^ ^]^
E 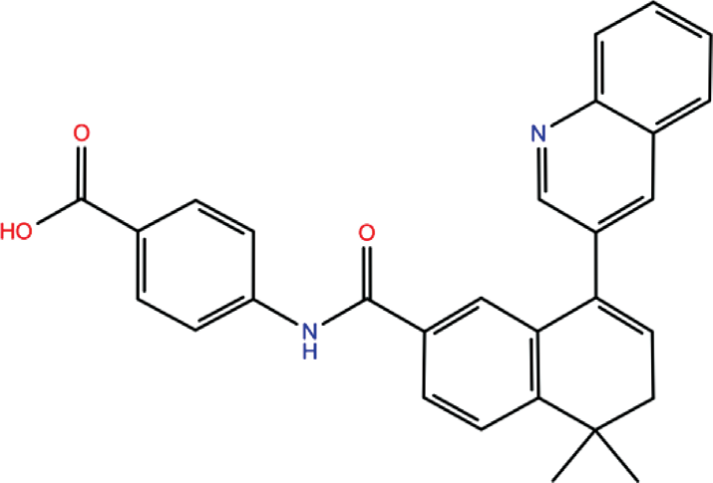	F 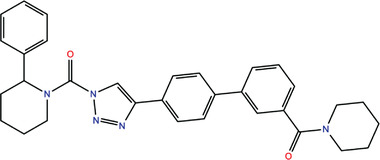
**BMS195614**: antagonist of the retinoic acid receptor (RAR)	**KT185**: inhibitor of α/β‐hydrolase domain‐containing 6 (ABHD6) in the brain and liver of mice
G 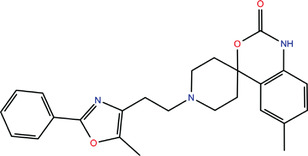	H 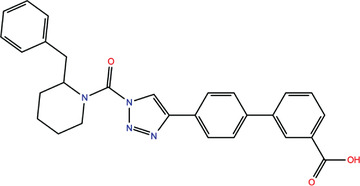
**RS504393**: antagonist of the CC2 chemokine receptor	**KT203**: inhibitor of ABHD6 activity in the liver of mice
I 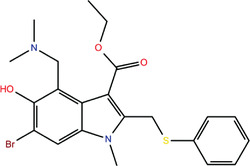	J 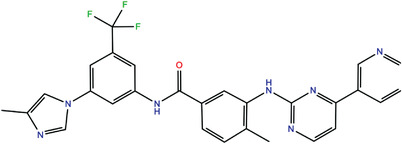
**Arbidol**: used as a treatment for influenza and other respiratory infections in Russia and China^[77]^	**Nilotinib**: a BCR‐ABL tyrosine kinase inhibitor used for the treatment of chronic myelogenous leukemia (CML)^[^ ^89^ ^]^

MD, molecular dynamics; RBD, receptor‐binding domain.

## PERSPECTIVES FOR TARGETING THE SPIKE PROTEIN

Although the worldwide effort to discover approved drugs to repurpose against the SARS‐CoV‐2 S protein, to date no MD‐based study has delivered working hypotheses resulting in clinical trials. Open access COVID‐19 drug repurposing databases^[^ ^90^, ^91^ ^]^ are a precious source of information but do not consider potential therapeutic agents proposed in silico; thus, there is a coordination gap between theoretical and experimental scientific communities.^[^ ^92^ ^]^

**Box 2**

From a technical perspective, the amount of MD sampling to confirm molecular docking predictions has been generally limited to the time scale of a few tens of nanoseconds and this has probably produced numerous in silico false positives, undermining the credibility of computation studies. Simulations over tens or few hundreds of nanoseconds showing a docking complex as stable should not be faithfully trusted. For example, a molecule with a residence time of few microseconds (way longer than usual MD post‐docking simulations) and an optimistic binding *k*_on_ of ≈10^7^ M^–1^ s^–1^ would have a kinetic affinity of about 10^–2^ M and therefore would not be a binder despite the indication provided by MD.

The discrepancy between the time scale of the real world and the simulated models (Box 2) can be partially overcome with end‐state methods such as the MM‐PBSA or MM‐GBSA,^[^ ^93^ ^]^ which can quantify the binding free energy using short MD simulations. However, the accuracy of these methods is system dependent and usually best suited for comparisons between congeneric ligands^[^ ^94^ ^]^ rather than very different chemotypes, as is usually required by repurposing strategies. Enhanced MD sampling techniques such as metadynamics^[^ ^95^ ^]^ speed up the time required to dissociate docking complexes and allow estimation of the stability of the bound ligand, and can therefore assist in recognizing docking false positives.

We screened in silico more than 2000 approved small molecules.^[^ ^78^ ^]^ After the docking and post‐docking MD simulations of the best‐ranked compounds docked in situ to the RBD, we performed a further step consisting of simulating the encounter of the RBD and ACE2 in the presence of the best compounds. Despite the stability displayed during the cefsulodin/RBD simulations, ACE2 easily displaced the proposed ligand in half of the supervised MD (SuMD)^[^ ^96^, ^97^ ^]^ replicas. Increasing the complexity of the modeled system highlighted the limit of common computational protocols for correctly selecting small molecules able to hinder the recognition between the S protein and ACE2. Therefore, dynamic approaches that consider the formation of the ternary systems between the S protein, ACE2, and the potential binding inhibitor should be routinely considered. Besides this, structure‐based drug repurposing strategies should take into account the fully glycosylated S protein and the discovery of allosteric sites on the RBD to overcome the targeting obstacles. The extreme flexibility of the glycans on the surface of the S protein and the effective steric hindrance they offer affect the ability of antibodies or potential therapeutic molecules to bind to a sufficiently exposed epitope.^[^ ^98^ ^]^ To combat this, Haji‐Ghassemi et al. suggested searching for drugs to target this shield^[^ ^99^ ^]^ this is a different approach from the traditional protein‐oriented one.

## MOLECULAR DYNAMICS INSIGHTS ON NEW SARS‐COV‐2 VARIANTS

Since the beginning of the pandemic, the evolutionary impact of SARS‐CoV‐2 was kept under observation by the scientific community to evaluate the possible effects of mutations on transmissibility, severity, and viral evasion of the immune mechanism.^[^ ^100^, ^101^, ^102^, ^103^ ^]^ Among the SARS‐CoV‐2 variants, the major preoccupations regarded those strains that carried important mutations and deletions, especially on the RBD (categorized as variants of concern [VOC]).^[^ ^100^ ^]^ Such VOC have important RBD mutations: B.1.1.7 (Alpha), carries E484K, N501Y, D614G, P681H; B.1.351 (Beta) carries K417N, E484K, N501Y, D614G, A701V; P1 (Gamma) carries K417T, E484K, N501Y, D614G, H655Y; B.1.617.2 (Delta) carries L452R, T478K, D614G, P681R.^[^ ^102^ ^]^ Concerns among the scientific community have risen due to their potential to elude the immune system and overcome vaccine protection^[^ ^104^, ^105^, ^106^ ^]^ despite showing an overall similarity between variants, which diverged only in terms of flexibility.^[^ ^107^ ^]^

More recently, a new B.1.1.529 (Omicron) VOC^[^ ^108^, ^109^, ^110^ ^]^ carrying N440K, G446S, S477N, 118 T478K, E484A, Q493R, G496S, Q498R, N501Y, and Y505H mutations, and its lineages became predominant over the Delta variant, possibly due to a more rapid entry or different mechanism,^[^ ^111^, ^112^, ^113^ ^]^ an enhanced ability to evade the immune system,^[^ ^103^, ^114^, ^115^ ^]^ and its increased affinity for ACE2^[^ ^116^, ^117^, ^118^ ^]^ although showing a milder pathogenic impact.^[^ ^119^ ^]^ New VOCs are expected to pose a new threat should they become widespread^[^ ^120^, ^121^ ^]^ and further studies should follow to evaluate the potential risk of new mutations.

MD‐based computational efforts evaluated the effect of omicron's mutations on ACE2 binding strength,^[^ ^118^, ^122^, ^123^ ^]^ suggesting that YG339D, N440K, S477N, T478K, Q493K, N501Y increase the binding affinity, as also reported by Socher et al.^[^ ^124^ ^]^ S371L, S373P, S375F, K417N, G446S, E484A, G496S, Q498R, Y505H, on the other hand, decreased the binding affinity for ACE2, in agreement with a compensatory effect that moderates the binding strength of the enhancing mutations.^[^ ^125^ ^]^ However, the reinforced network of hydrogen bonds, involving T500‐D355, G502‐K353, N487‐Y83, as well as R493‐D38, and A475‐S19, paired with the electrostatic matching between R493‐D38 and the loop shift caused by E484A and T478K mutation as suggested by Zhao et al.,^[^ ^126^ ^]^ suggesting an overall increase in the binding energy. These shifts seem to increase the complementarity between ACE2 and Omicron's RBD and could be the reason for the increased binding affinity, as also highlighted by Nie et al.^[^ ^127^ ^]^

The advent of the new VOC highlighted the necessity to follow multiple paths, for a broad‐spectrum therapeutic approach, which should not only consider RBD as the target of main interest but should also consider more conserved viral proteins among the variants. MD studies were carried out to investigate non‐structural proteins (NSP) as potential druggable targets:^[^ ^128^, ^129^ ^]^ Vivek et al. suggested the NSP13 helicase ATP‐binding sites as a druggable target, while Vardhan et al. included the NSP14 and NSP15 exonuclease and endonuclease, respectively. Both groups used phytochemical small molecules as target binders, which, however, still require experimental data to confirm their efficacy.

Alternatively, PF‐07321332 a promising oral antiviral candidate against the main protease (MPro) catalytic dyad on residues His41‐Cys145 was investigated using MD simulation by Macchiagodena et al., using preliminary data structures available.^[^ ^130^ ^]^ According to Macchiagodena's work, the formation of the thiolate‐imidazolium, paired with the exposition of the nitrile warhead in the proximity of the Cys145 would allow for the electrophilic attack on the MPro, for effective enzyme inhibition.

However, MD is not the most adequate method to represent bond formation or breaking, but the insights provided by contact frequency and interatomic distances could support the description of the experimental data, once the PF‐07321332‐MPro complex structure will become publicly available.

Other attempts against MPro were done^[^ ^130^, ^131^, ^132^, ^133^ ^]^ but require further experimental data to validate the hypothesis. The RNA polymerase,^[^ ^134^ ^]^ as well as the nucleocapsid,^[^ ^135^ ^]^ and envelope protein^[^ ^136^ ^]^ were proposed as a druggable target, but these studies will require further confirmation by experimental data to verify whether NSPs could be considered viable targets.

## CONCLUSION

As a state‐of‐the‐art computational technique, MD has been broadly employed to interrogate the structure and function of the S protein at the atomic level to understand how its inherent flexibility modulates the binding to ACE2 receptors and, therefore, SARS‐CoV‐2 virulency. MD suggested unexpected flexibility of the stalk region S2, the role of glycans on the S protein surface, and the contribution of single residues on the RBD to the interactions with ACE2. MD contributed important dynamic and structural elements such as the minimum angulation required for molecular recognition between ACE2 and RBD, the effects of mutations on the binding capacity of the S protein, and the structural and protective role of glycans. Through MD, it was possible to understand the spontaneous motions that open the RBD from the “down” to “up” conformation, revealing numerous cryptic pockets, which are possible targets of new drugs. The “down” to “up” transition that the RBD undergoes before ACE2 recognition was another important phenomenon MD delivered structural insights on.

From a future perspective, we believe there is scope for an increasingly important contribution of MD in the study of Ab and their rational development as therapeutic agents. Also, MD contributed to rationalizing in vitro data on potential S protein binding antagonists, but with limited utility in drug repurposing. Approaches to address COVID‐19 start to fade away from drug repurposing and the S protein to more classic rational strategies to target functional viral proteins, as demonstrated by the main protease (Mpro) inhibitor nirmatrelvir, the first oral anti‐COVID‐19 drug approved by the FDA. In this scenario, it is plausible that MD will regain a central role in aiding the development of future new classes of therapeutics against SARS‐CoV‐2.

## CONFLICT OF INTEREST

The authors do not have any conflicts of interest to declare.

## Data Availability

The data described in this review was obtained from the cited articles.
